# Versatile Photo/Electricity Responsive Properties of a Coordination Polymer Based on Extended Viologen Ligands

**DOI:** 10.3390/membranes12030277

**Published:** 2022-02-28

**Authors:** Xiaohan Peng, Yuchen Shi, Zhiqiang Zeng, Jianming Zheng, Chunye Xu

**Affiliations:** Hefei National Laboratory for Physical Sciences at the Microscale, Department of Polymer Science and Engineering, University of Science and Technology of China, Hefei 230026, China; xhpeng@mail.ustc.edu.cn (X.P.); shiyc@mail.ustc.edu.cn (Y.S.); zengzq@mail.ustc.edu.cn (Z.Z.); jmz@ustc.edu.cn (J.Z.)

**Keywords:** coordination polymer, multi−responsive, photochromism, photomodulated fluorescence, electrochromism, electrofluorochromism

## Abstract

Responsive chromogenic materials have attracted increasing interest among researchers; however, up until now, few materials have exhibited multifunctional chromogenic properties. The coordination polymers (CPs) provide intriguing platforms to design and construct multifunctional materials. Here, a multifunctional photo/electricity responsive CP named Zn−Oxv, which is based on the “extended viologen” (ExV) ligand, was synthesized. The Zn−Oxv exhibited reversible photochromism, photomodulated fluorescence, electrochromism and electrofluorochromism. Furthermore, we prepared Zn−Oxv thin films and investigated electrochromic (EC) properties of viologen−based CPs for the first time. Zn−Oxv thin films showed excellent EC performance with a rapid switching speed (both coloring and bleaching time within 1 s), high coloration efficiency (102.9 cm^2^/C) and transmittance change (exceeding 40%). Notably, the Zn−Oxv is by far the fastest CP EC material based on redox−active ligands ever reported, indicating that the viologen−based CPs could open up a new field of materials for EC applications. Therefore, viologen−based CPs are attractive candidates for the design of novel multi−responsive chromogenic materials and EC materials that could promise creative applications in intelligent technology, dynamic displays and smart sensors.

## 1. Introduction

Smart responsive chromogenic materials that exhibit changes in light absorption or emission properties under external stimuli have been investigated extensively in recent years [1,2,3,4]. Among these chromic materials, optically and electrochemically active chromic materials promise attractive applications in sensors, lighting, optoelectronic devices and smart technology devices [5,6,7]. A number of photo/electro−responsive chromogenic materials, such as electrochromic (EC), photochromic and electrofluorochromic (EFC) materials have already been reported [8,9,10,11,12,13]. Unfortunately, most of them are mono−responsive, which restricts their practical applications.

Electron−deficient 1,1′−disubstituted−4,4′−bipyridinium derivatives (viologens) are known for their capability of distinct optical displays. With the assistance of appropriate electron donors, they can undergo reversible redox reaction via electron transfer (ET) and exhibit three redox states (dication (V^2+^), radical cation (V^+^) and neutral (V^0^)), which results in the change of optical properties [14,15]. Due to these special capabilities, viologen derivatives can be used as photo/electro−responsive materials. However, most viologen derivatives are electro−responsive but non−photoresponsive. The coordination polymers (CPs) constructed from metal clusters and organic ligands could provide cooperation between components and the inter−contact structural framework, which can create effective channels for ET, allowing for the transition of viologen units between different redox states under stimuli [16,17,18,19,20]. As a consequence, taking advantage of the great versatility afforded by coordination chemistry, nonphoto−responsive viologen ligands may not only result in photo−responsiveness, but could also integrate photochemical and electrochemical functionalities [20,21]. The viologen derivatives, which consist of two pyridinium rings spaced by conjugating aryl groups, named “extended viologen” (ExV), display similar redox features to the viologen units [20]. According to previous studies, the ExV could afford new fluorescence properties [22]. Therefore, ExV−based CPs are expected to realize the coupling of electro/photo−chromism with fluorescence properties. Viologens are typical organic EC materials with a dramatic optical contrast and have been widely explored. However, viologens are common solution−type EC materials, which cannot be bleached by applying a reverse voltage but only by write−erase effect, resulting in a long bleaching time [23,24]. Viologen−based CPs can be film−forming as a way of solving this problem. Moreover, CPs possess tunable redox−active sites, surface areas and compositions, which are favorable factors as EC materials [25,26]. Therefore, viologen−based CPs are promising EC materials, but their EC properties have rarely been investigated.

**Figure 1:**
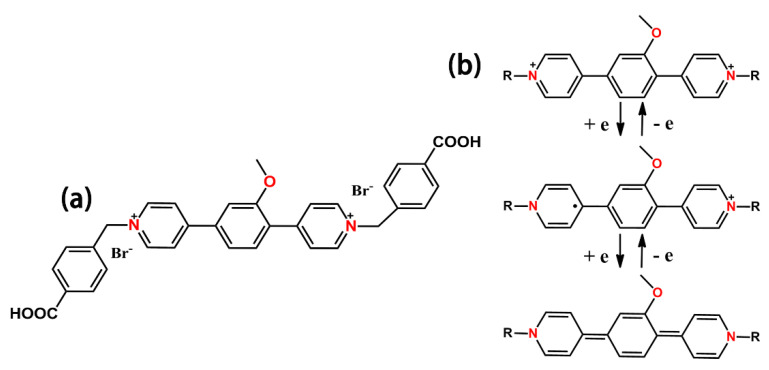
(**a**) The chemical structure of Oxv. (**b**) Stepwise reduction of the Oxv unit.

In this research, we synthesized a new “extended viologen” (ExV) ligand named (2−methoxy−1,4−phenylene) bis(1−carboxybenzy)−4,4’−bipyridinium dibromide (Oxv). As shown in Figure 1, the Oxv ligand can also undergo a two−step reversible redox process. Furthermore, we chose *p*−benzenedicarboylic acid (*p*−H_2_BDC) as the second ligand and d^10^ Zn ion to construct electron−rich ZnO−carboxylates to supply electrons to electron−deficient Oxv. Finally, we obtained a multifunctional photo/electricity responsive CP named Zn−Oxv which can achieve the photo/electro−modulated chromism and luminescence. Photochromism and photomodulated fluorescence of the Zn−Oxv were further discussed. Additionally, we fabricated the viologen−based CP thin films for the first time and investigated its EC−EFC properties. The Zn−Oxv films exhibited excellent EC performance as potential EC materials.

## 2. Materials and Methods

### 2.1. Materials

All solvents and chemicals used in this research were commercial products. Ethanol, N, N−dimethylformamide (DMF), methanol, chloroform, ethyl acetate, acetone, acetonitrile, zinc chloride (ZnCl_2_), *p*−benzenedicarboylic acid (*p*−H_2_BDC), propylene carbonate (PC), ethanol, lithium perchlorate (LiClO_4_) and potassium carbonate were purchased from Sinopharm Chemical reagent Co. Ltd. (Shanghai, China), 2,5−Dibromoanisole, 4−pyridineboronic acid, (beta−4)−platinum and tris(dibenzylideneacetone)dipalladium(0), 4−Bromomethylbenzoic acid, and 1,4−Dioxane were bought from Energy Chemical (Shanghai, China).

### 2.2. Methods

Nuclear magnetic resonance (NMR) spectra were recorded on a Bruker Avance AV400 instrument (400 MHz). Mass spectrum was examined by a Thermo Fisher Scientific ProteomeX−LTQ mass spectrometer (Heifei, China). The Fourier transform infrared (FTIR) spectra of Zn−Oxv compound and the Oxv free ligand were implemented using a Bruker Vector 22 spectrometer in the range of 400−4000 cm^−1^. The morphology of Zn−Oxv powder and Zn−Oxv films was observed by using a GeminiSEM 500 Schottky field emission scanning electron microscope. X−ray photoelectron spectroscopy (XPS) measurements of the Zn−Oxv compound were carried out using an ESCALAB 250 spectrometer (Thermo−VG Scientific, East Grinstead, West Sussex, UK), where Al−Kα was used as the X−ray source. Electron spin resonance (ESR) signals of the Zn−Oxv compound were recorded by JES−FA200 at room temperature. Solid−state UV−vis spectra of Zn−Oxv powder were recorded on a SOLID3700 UV–vis–NIR spectrophotometer. Photoluminescence (PL) spectra were examined by a JOBIN YVON Flurolog−3−TAV fluorescence spectrophotometer. The cyclic voltammetry (CV) tests of Zn−Oxv thin films were conducted by a three−electrode system using a CHI 660D, which included the ITO substrate (0.7 × 3.0 cm^2^) with Zn−Oxv thin films, platinum sheet and the silver wire as the working electrode, the counter electrode and the reference electrode. The spectroelectrochemical analysis of Zn−Oxv films was performed by the combination of the CHI 660D electrochemical analyzer and JASCO V−670 UV–vis–NIR spectrophotometer. Electromodulated fluorescence spectra of Zn−Oxv films were examined by the combination of CHI 660D electrochemical analyzer and JOBIN YVON Flurolog−3−TAV fluorescence spectrophotometer.

### 2.3. Synthesis of the ExV Ligand

The 2, 5−di (4−pyridyl) anisole was synthesized according to the previously reported procedure in the literature [23]. Alkylation reaction of 2, 5−di (4−pyridyl) anisole was produced by reacting with 4−bromomethylbenzoic acid in acetonitrile. The mixture was heated at 90 °C with reflux and stirred for 2 days. The precipitate was filtered and washed repeatedly with acetonitrile. 

^1^H NMR spectrometry data are presented below:

2, 5−di (4−pyridyl) anisole: ^1^H NMR (300 MHz, CDCl_3_–*d_6_*, ppm): δ 8.715–8.692 (d, 2H), δ 8.671–8.648 (d, 2H), δ 7.563–7.542 (d, 2H), δ 7.523–7.501 (d, 2H), δ 7.483–7.455 (d, 3H), δ 3.942–3.925 (s, 3H).

(2−methoxy−1,4−phenylene)bis(1−carboxybenzy)−4,4’−bipyridinium dibromide (Oxv): ^1^H NMR (300 MHz, DMSO−*d_6_*, ppm): δ 9.394–9.359 (d, 2H), δ 9.296–9.255 (d, 2H), δ 8.769–8.732 (d, 2H), δ 8.769–8.732 (d, 2H), δ 8.048–7.999 (d, 4H), δ 7.927–7.854 (m, 3H), δ 7.732–7.666 (m, 4H), δ 6.037–5.973 (d, 4H), δ 4.059–4.010 (s, 3H).

### 2.4. Preparation of Zn−Oxv Powder and Zn−Oxv Thin Films on ITO Substrates

A combination of 2.5 × 10^−2^ mmol Oxv, 2.5 × 10^−2^ mmol ZnCl_2_ and 2.5 × 10^−2^ mmol p−H_2_BDC was mixed in CH_3_OH−DMF (20.0 mL, *v*/*v* = 1/3) and stirred for 10 min. A total of 5 mL resulting solution was added into a 25 mL Teflon−lined stainless−steel reactor containing a vertical ITO glass; the above reaction was maintained at 120 °C for 3 h. After cooling to room temperature, the yellow Zn−Oxv powder at the bottom of the reactor was isolated, rinsed by H_2_O and ethanol three times and dried in air. Moreover, the prepared films were removed from the solution and washed briefly with ethanol to remove powder residual. Bare ITO glass substrates (0.7 × 3.0 cm^2^) were ultrasonically cleaned with acetone, methanol and deionized water, in turn, and dried in a vacuum afterwards.

## 3. Results

### 3.1. Structure Verification

**Figure 2:**
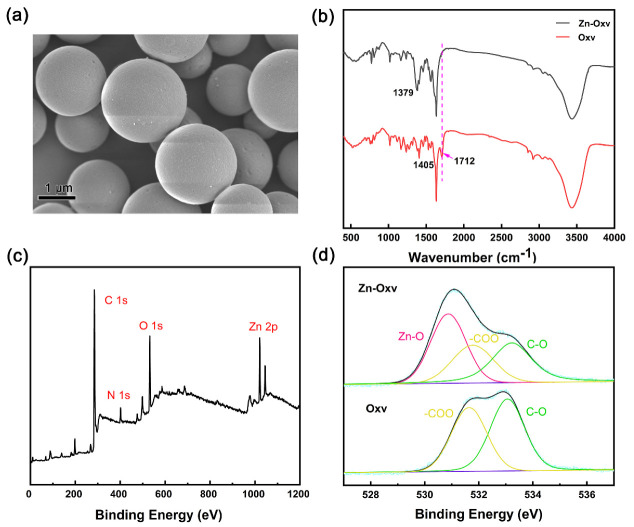
(**a**) The SEM image of Zn−Oxv. (**b**) FTIR spectra of the Zn−Oxv compound and free ligand (Oxv). (**c**) Broad XPS spectrum of the Zn−Oxv compound. (**d**) High−resolution XPS spectra of O 1s for Zn−Oxv.

Zn−Oxv CPs were synthesized via a hydrothermal method; the SEM image showed a regular spherical morphology of Zn−Oxv (Figure 2a). For the FTIR spectra of Zn−Oxv (Figure 2b), the strong absorption bands, which correspond to the vibration of the phenyl ring observed, confirm the presence of the Oxv ligand [27]. Additionally, for the stretching vibration of carbonyl, the representative absorption band around 1700 cm^−1^ disappeared; the absorption band around 1400 cm^−1^ exhibited a bathochromic effect, indicating the coordination interaction between the zinc center and the carboxyl group in the Zn−Oxv [28,29]. XPS measurements were carried out to examine the element compositions and to record the valence state changes in the desired Zn−Oxv CPs; the survey spectrum is exhibited in Figure 2c. The high−resolution spectrum of O 1s can be deconvoluted into peaks at 530.8, 531.8 and 533.2 eV, shown in Figure 2d, which can be assigned to Zn−O, −COO and C−O [30,31]. In comparison with O 1s of the Oxv ligand, the presence of the Zn−O bond in Zn−Oxv CPs further confirms the coordination interaction. The characteristic binding energy of Zn 2p_3/2_ and 2p_1/2_ is observed at 1021.9 and 1045.0 eV (Figure S8), respectively, which is consistent with the coordination environment of the zinc and the oxygen atoms [31]. The thermal stability of Zn−Oxv was proven by thermogravimetric analysis. The collapse of the whole structure happened when the temperature reached 260 °C, as shown in Figure S9. All characterization results showed successful fabrication of Zn−Oxv CPs.

### 3.2. Multi−Stimuli Responsive Properties

#### 3.2.1. Photochromism and Photomodulable Fluorescence

**Figure 3:**
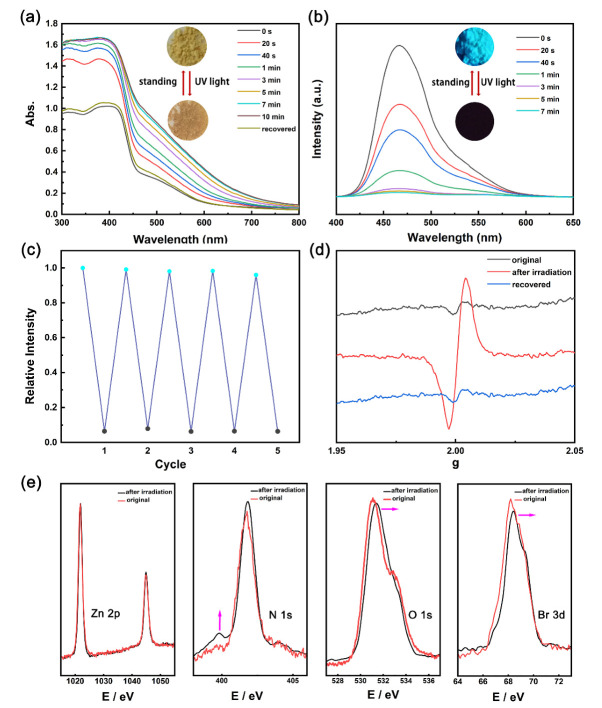
(**a**) Solid−state UV−vis spectra show the photochromic behavior of the Zn−Oxv compound. The inset shows the color change of Zn−Oxv powder. (**b**) The emission spectra of the Zn−Oxv compound changed during UV light illumination. The inset shows disappearance of visible emission. (**c**) Five cycles of fluorescence on−off switching at λ = 467 nm. The emission intensity of recovery state (blue point) and stimulated state (black point). (**d**) ESR spectra for the Zn−Oxv compound before, after irradiation and recovered in the solid state at room temperature. (**e**) High−resolution XPS spectra of Zn 2p, N 1s, O 1s and Br 3d for the Zn−Oxv compound before and after irradiation.

Non−photoresponsive viologen units may result in photo−active CPs via photo−induced electron transfer (PET) through interpenetrating structure. When the n−Oxv compound was exposed to UV light, the photo−responsive phenomenon also occurred. Zn−Oxv powder can realize visible color change from yellow to brownish red under UV irradiation; the solid UV–vis–NIR spectrophotometer records the process. A series of spectra demonstrated that the prolonged irradiation caused a continuous increase in absorption intensity over the wavelength from 300 to 700 nm (Figure 3a). Furthermore, a visible color change occurred within 20 s of UV irradiation, indicating the rapid responsive rate, which is favorable for practical applications. The ExV ligand Oxv exhibited intensive cyan fluorescence emission; the Zn−Oxv compound also displayed intensive fluorescence emission around 466 nm (Figure S10). Under the UV light, the emission intensity of Zn−Oxv also dropped dramatically along with the color change. The intensity dropped by 40% after 20 s and reached saturation after 3 min. The fluorescence decay also showed a high responsive rate. Zn−Oxv compound returned to its original state after around several days at ambient environment. This reversible process can be repeated over five times without eye−detectable color loss and obvious fluorescence intensity changes (Figure 3a,c). Additionally, the solid−state UV−vis spectrum and fluorescence spectrum of the Oxv ligand exhibited no obvious change before and after UV light irradiation, further demonstrating that the photo−responsive effect originated from the coordination structure (Figures S11 and S12).

To characterize the mechanism of the photo−responsive process of the Zn−Oxv compound, ESR measurements were performed (Figure 3d). After irradiation, the Zn−Oxv showed a strong single−line signal centered at around g = 2.0006, which is very close to those reported free radicals [32,33]. No ESR signal of Zn−Oxv at original state and after recovery was observed. As previous research reported, the radical species can result in color change and fluorescence quenching. Therefore, the results of ESR measurements indicate that the formation of radical species via PET is responsible for photochromism and photomodulable fluorescence.

To gain further insight into the pathway of PET, XPS spectra of the Zn−Oxv powder were recorded before irradiation and upon irradiation for 5 min (Figure 3e). Compared with the original state, the Zn 2p core−level band had no significant change, but the XPS spectra of N 1s, O 1s and Br 3d showed obvious shifts. A new lower−energy band of N 1s appeared at 399.7 eV after irradiation, implying N atoms in pyridinium units are the electron acceptors [34]. The spectrum of O 1s before irradiation exhibited a peak at 531.0 eV and the peak shifted to higher energy after irradiation, suggesting some oxygen atoms were served as electron donors. As mentioned above, the Zn−Oxv compound showed a fast photo−response, while the other reported viologen−based photo−responsive materials usually required longer time [19,35]. This fast speed of Zn−Oxv may be mainly attributed to the fact that Zn−carboxylate clusters possess inherent abundance of O atoms, excellent stability and redox activity, facilitating the formation of ET pathways [18,35,36]. In addition, the peak of Br 3d core−level moved to a higher energy, from 68.1 to 68.4 eV, demonstrating that a portion of the bromide ions also served as electron donors. FTIR spectra of Zn−Oxv with no difference compound before and after irradiation are shown in Figure S13.

#### 3.2.2. Electrochromism and Electrofluorochromism

**Figure 4:**
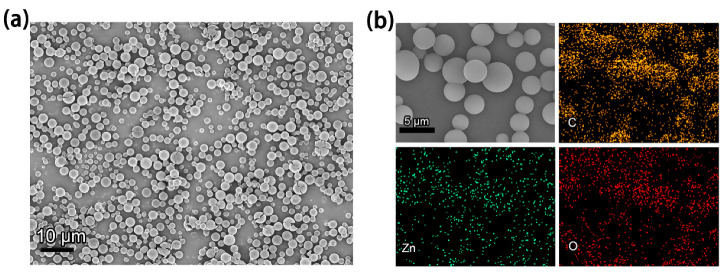
(**a**) The SEM image and (**b**) Elemental mapping of the Zn−Oxv thin film.

In this research, we fabricated Zn−Oxv films on ITO substrates to investigate the electro−responsive property. As seen in SEM images and elemental mapping of the samples (Figure 4a,b), the Zn−Oxv thin film consisted of spherical Zn−Oxv CPs and results revealed the presence of C, O, Zn that correspond to the main constituents of the Zn−Oxv, showing successful fabrication of Zn−Oxv CPs films on the ITO substrate. The cyclic voltammetry (CV) of Zn−Oxv films utilizing the three−electrode system was measured. The system included 0.2 M PC/LiClO_4_ solution as the electrolyte, a Zn−Oxv film on the ITO substrate (0.7 × 3.0 cm^2^) as the working electrode, the platinum plate as the counter electrode and the silver wire as the reference electrode. The resultant CV with a potential window of −1.3 to 0 V is presented inFigure 5a. Herein, the obtained CV revealed a one−step two−electron reduction process or two very closely spaced one electron reduction processes of the Zn−Oxv compound, which is similar to other reported extended viologens [23,37,38].

**Figure 5:**
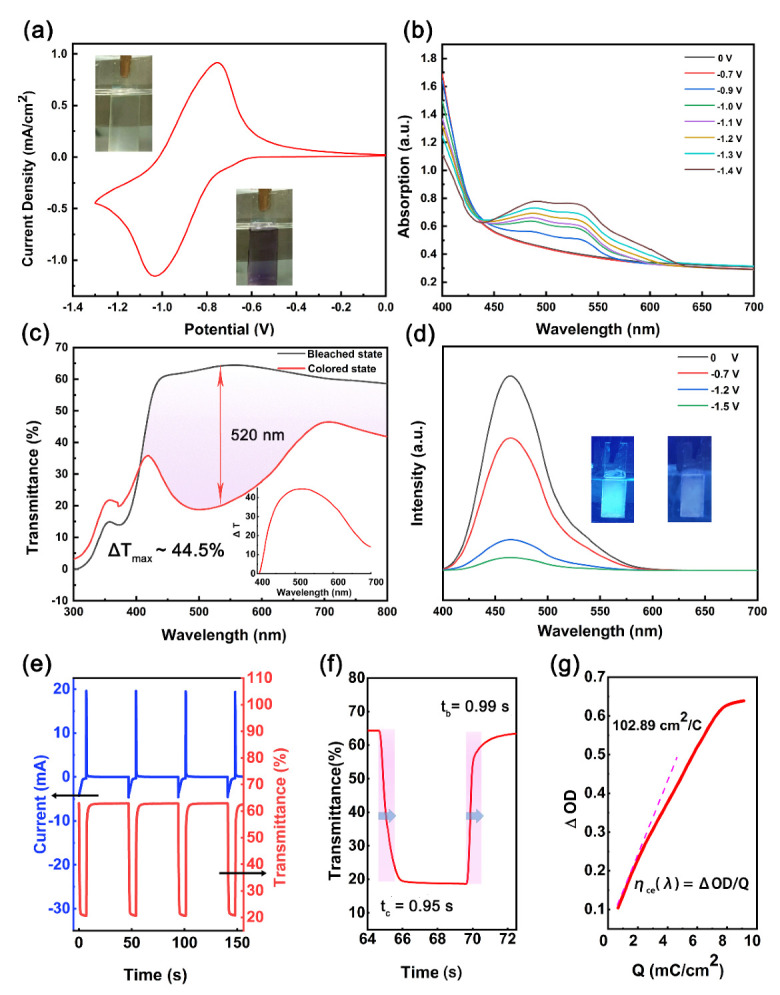
(**a**) The cyclic voltammogram of Zn−Oxv films (scan rate of 100 mV s^−1^). (**b**) Spectroelectrochemical spectra of Zn−Oxv films under different external potentials. Inset: photographs of Zn−Oxv thin films at colored and bleached states. (**c**) The transmittance spectrum of Zn−Oxv films at colored and bleached states. Inset: transmittance differences between colored and bleached states. (**d**) Fluorescence intensity differences between the fluorescent and nonfluorescent states. Inset: photographs of Zn−Oxv films at fluorescent and nonfluorescent states. (**e**) Variation of current and corresponding transmittance switching spectra (recorded at λ = 520 nm) with applied square wave used for color switching of Zn−Oxv thin films. (**f**) Transmittance switching between colored and bleached state. (**g**) The coloration efficiency of Zn−Oxv thin films.

EC materials display dynamic optical−switching via electrical stimuli. The color of the Zn−Oxv films also changes during the electrochemical reduction process and spectroelectrochemical spectroscopy can monitor the color change. The spectroelectrochemical analysis of Zn−Oxv films was performed by the combination of the electrochemical analyzer and spectrophotometer, using the same three−electrode system as the CV test. A series of absorbance spectra are shown in Figure 5b. Stepwise applied potentials from −0.7 V to −1.4 V caused an increased absorption from 440 to 550 nm, while turning the color of the films from pale yellowish green to purple. In addition, the applied potentials at −1.4 V gave rise to a maximum light transmittance change (Δ*T*) exceeding 40% around 520 nm (Figure 5c). Δ*T* was determined as *T*_b_ (λ) − *T*_c_ (λ), where *T*_b_ and *T*_c_ refer to light transmittance in bleached and colored states at a specific wavelength, respectively. Interestingly, due to the fluorescent ExV ligands, not only the color of the Zn−Oxv film changed with increasing potential, but the fluorescence emission intensity also dropped. The spectra of fluorescence intensity were tested via the same three−electrode system by the combination of a fluorescence spectrophotometer using an electrochemical analyzer (Figure 5d). Zn−Oxv films returned to a bleached state and a fluorescent state by applying a reverse potential. Both the color change and fluorescence switching were attributed to the electrochemical reduction of viologen units that led to the increased absorption in the visible region and effective fluorescence quenching [14]. The Zn−Oxv thin film showed EC−EFC bifunctional performance, enabling its extensive applications in both bright and dark conditions.

The response time and coloring efficiency (CE), which are important parameters for EC properties, were tested by the combination of UV–vis spectrophotometer and double−step chronoamperometry techniques using the three−electrode system. The final variation of current and corresponding transmittance switching spectra (recorded at λ = 520 nm) under applied square wave potential (−1.2 V applied for 7 s and 1.5 V applied for 40 s) used for complete color switching are illustrated in Figure 5e. The switching speed was defined as the time period required for achieving 90% of the transmittance change in a specific wavelength. The calculated coloration time (*t*_c_) and bleaching time (*t*_b_) were both approximately 1 s, showing rapid response speed (Figure 5f). This phenomenon can be attributed to the high electrochemical response activity of viologen derivatives. On the other hand, the large contact area between spherical Zn−Oxv particles and electrolytes increased the active sites for redox reactions. Another criterion of EC performance is the CE, defined as CE = Δ OD/(*Q*/*A*) = log (*T_b_*/*T_c_*)/(*Q*/*A*), wherein ΔOD denotes variation in optical density, (*Q/A*) is the intercalated charge per unit area, and *T*_b_ and *T*_c_ refer to transmittance values at the bleached and colored state at a certain wavelength. The CE value can be calculated by the variation of current and transmittance switching spectra. The relationship between the optical change at 520 nm and unit charge density is shown in Figure 5g; the value of CE was 102.9 cm^2^/C. Because organic ligands are mostly redox−inactive, electrochromic CPs based on functional redox ligands have rarely been reported. The Zn−Oxv thin films in this work exhibited the fastest response speed, a high Δ*T* and CE value compared with reported CPs based on other redox functional ligands (Table 1), indicating that viologen−based CPs are potential candidates for EC materials.

## 4. Conclusions

In summary, a multi−responsive chromogenic CP based on ExV ligands with properties of photochromism, photomodulated fluorescence, electrochromism and electrofluorochromism was synthesized. Under UV irradiation, the Zn−Oxv powder displayed a reversible color change from yellow to brownish red, along with quenching and recovery of fluorescence. The aforementioned results demonstrate that such a photo−responsive process originates from the formation of free nitrogen radicals through PET. Furthermore, the EC−EFC properties of Zn−Oxv were investigated for the first time. The Zn−Oxv films showed a reversible color switch between pale yellowish green and purple under applied potential; the color change was accompanied by fluorescence quenching and recovery. The Zn−Oxv films exhibited a rapid switching speed (both coloring and bleaching time within 1 s), high coloration efficiency (102.9 cm^2^/C) and high transmittance change (exceeding 40%), indicating that the CPs based on viologen ligands are candidates for EC materials. In brief, extended work along with the viologen−based CPs is expected to develop various multi−responsive chromogenic materials and EC materials relevant to smart technologies due to tunable redox activity and high charge−deficient characteristic.

## Figures and Tables

**Table 1:** Comparison of EC properties of Zn−Oxv films and other CP films based on functional ligands.

Compounds	Functional Ligands	Δ*T* (%)	*t*_c_ (s)	*t*_b_ (s)	Reference
Zn−Oxv	viologen	44.5	0.95	0.99	This work
Zn−NDI−74	NDI	21	3	91	[36]
Cu_3_(HHTP)_2_	triphenylene	40	3.2	5.9	[37]
NU−901	pyrene	62	12	5	[38]
CuTCA	TCA	65	4.8	3.3	[39]
Zn−MOF−74	DOBDC	13	8	9	[40]
Ni−MOF−74	DHTA	44.4	24.5	23.5	[41]

Δ*T* (transmittance change), *t*_c_ (coloration time), *t*_b_ (bleaching time), NDI (Naphthalenediimide), TCA (4,4′,4″−tricarboxytriphenylamine), DHTA (2,5−dihydroxyterephthalic acid), DOBDC [tris(hydroxymethyl)aminomethane].

## Data Availability

Data sharing is not applicable to this article.

## Supplementary Materials

The following supporting information can be downloaded at: https://www.mdpi.com/article/10.3390/membranes12030277/s1, Figure S1: The synthetic route of (2−methoxy−1,4−phenylene)bis(1−carboxybenzy)−4,4′−bipyridinium dibromide (Oxv). Figure S2: 1H NMR spectrum of 2,5−Di (4−pyridyl) anisole in CDCl3. Figure S3: 1H NMR spectrum of 2,5−Di (4−pyridyl) anisole in CDCl3. Figure S4: CNMR spectrum of the Oxv ligand. Figure S5: The FTIR spectrum of the Oxv ligand. Figure S6: The MS spectrum of the Oxv ligand. Figure S7: The TGA−DSC spectrum of the Oxv ligand. Figure S8: High−resolution XPS spectra of Zn 2p, C 1s and N 1s for the Zn−Oxv. Figure S9: The TGA curve of Zn−Oxv CPs. Figure S10: Fluorescence spectra of Zn−Oxv sample. Figure S11: Solid−state UV−vis spectra of free ligand (Oxv) before and after irradiation. Figure S12: Emission spectra of free ligand (Oxv) before and after irradiation. Figure S13: FTIR spectra of Zn−Oxv compound before and after irradiation.
