# Supplementary material for: Versatile Photo/Electricity Responsive Properties of a Coordination Polymer Based on Extended Viologen Ligands
Source: Membranes (Basel). 2022 Feb 28;12(3):277. doi: 10.3390/membranes12030277 (PMC8955544; doi:10.3390/membranes12030277)
Supplement: Supplementary file 1 [file membranes-12-00277-s001.zip › membranes-1584929-supplementary.pdf]

Supporting Information

Versatile Photo/electricity Responsive Chromogenic Properties of a Coordination Polymer Based on Extended Viologen Ligands

Xiaohan Peng, Yuchen Shi, Zhiqiang Zeng, Jianming Zheng & Chunye Xu*

*Corresponding Author：Chunye Xu

*E-mail: chunye@ustc.edu.cn

Website: http://www.hfnl.ustc.edu.cn/2010/0819/954.html

Address: Hefei National Laboratory for Physical Sciences at the Microscale, CAS Key Laboratory of Soft Matter Chemistry, Department of Polymer Science and Engineering, University of Science and Technology of China, Hefei 230026, P.R. China.

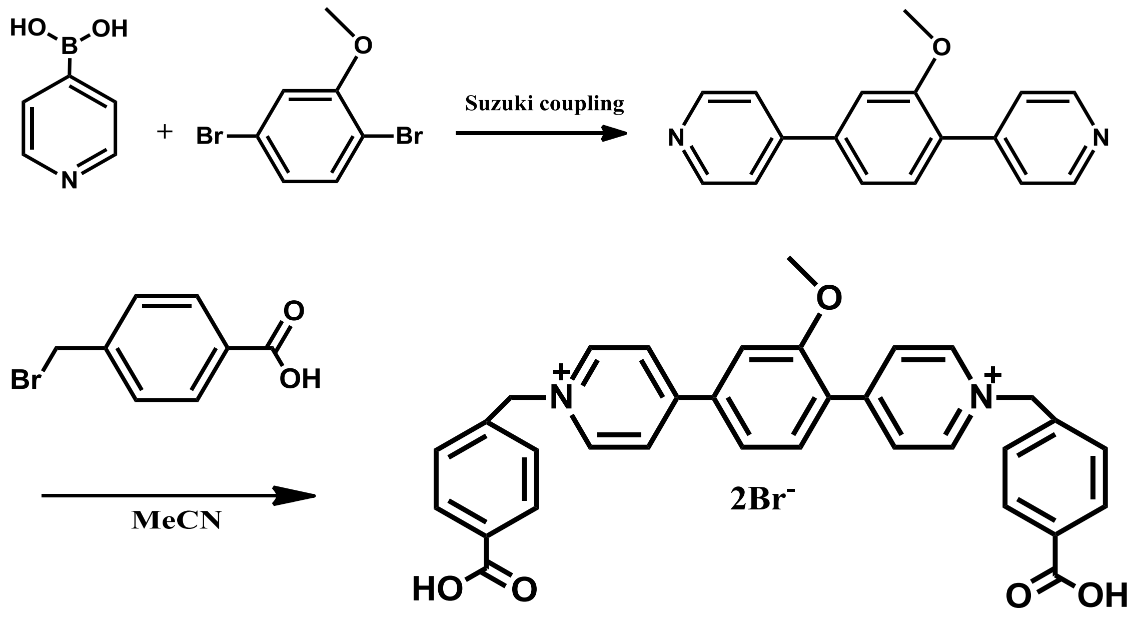

Figure S1. The synthetic route of (2-methoxy-1,4-phenylene)bis(1-carboxybenzy)-4,4'-bipyridinium dibromide (Oxv).−

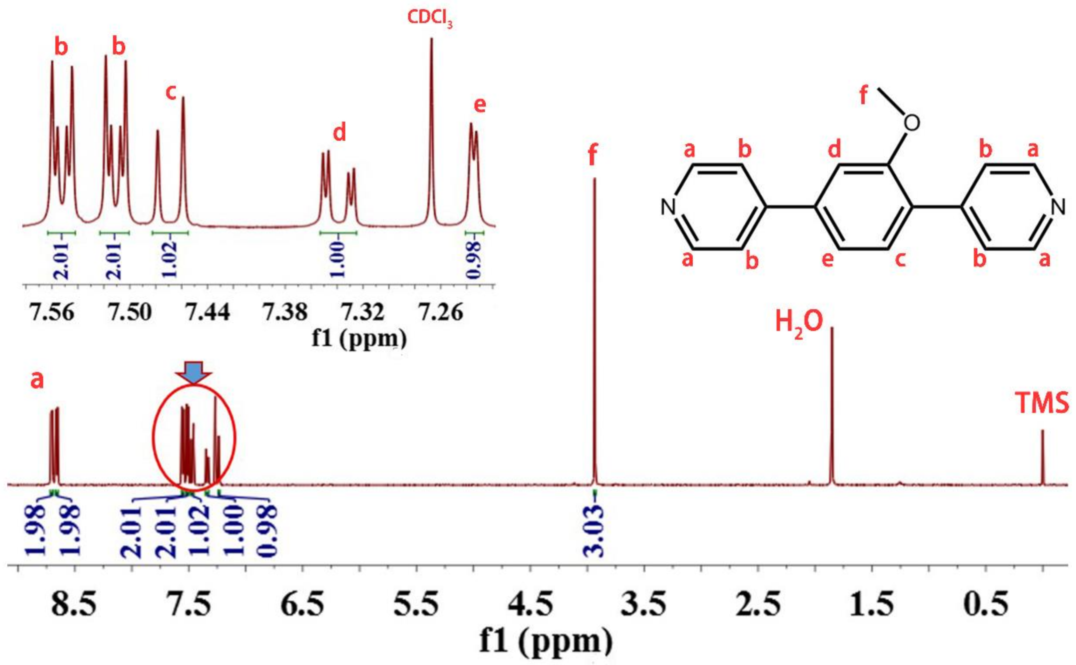

Figure S2. ^{1}H NMR spectrum of 2, 5-Di (4-pyridyl) anisole in $CDCl_3$.

^{1}H NMR (300 MHz, CDCl3–d6, ppm): δ 8.715–8.692 (d, 2H), δ 8.671–8.648 (d, 2H), δ 7.563–7.542 (d, 2H), δ 7.523–7.501 (d, 2H), δ 7.483–7.455 (d, 3H), δ 3.942–3.925 (s, 3H).

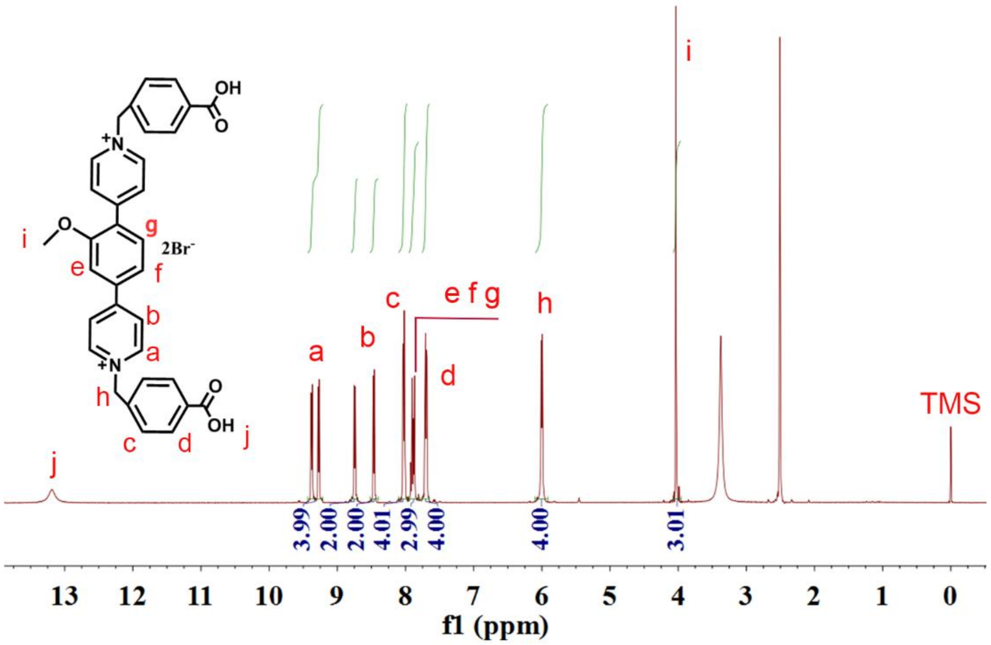

Figure S3. ^{1}H NMR spectrum of 2, 5-Di (4-pyridyl) anisole in $CDCl_3$.

^{1}H NMR (300 MHz, DMSO−d6, ppm): δ 9.394–9.359 (d, 2H), δ 9.296–9.255 (d, 2H), δ 8.769–8.732 (d, 2H), δ 8.769–8.732 (d, 2H), δ 8.048–7.999 (d, 4H), δ 7.927–7.854 (m, 3H), δ 7.732–7.666 (m, 4H), δ 6.037–5.973 (d, 4H), δ 4.059–4.010 (s, 3H).

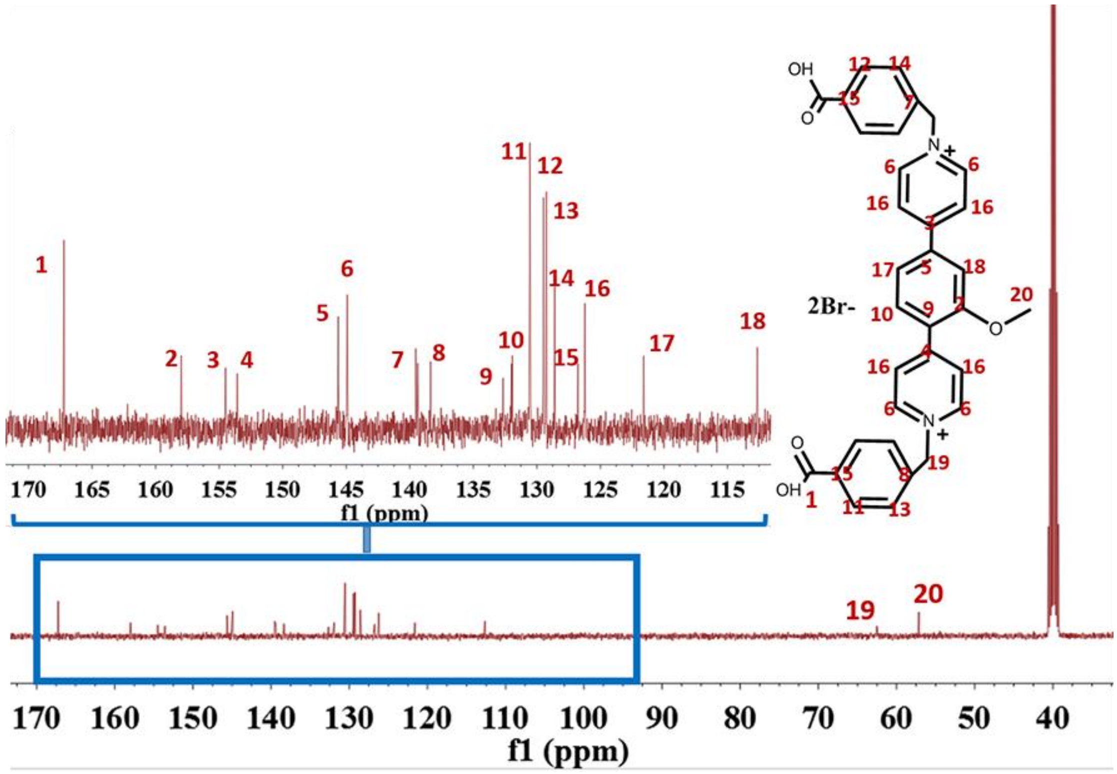

Figure S4. CNMR spectrum of the Oxv ligand.

^{13}C NMR (75 MHz, DMSO-d_6, ppm): 167.28, 158.06, 154.54, 153.62, 145.66, 144.96, 139.54, 138.42, 132.64, 132.07, 130.61, 129.55, 129.27, 128.63, 126.73, 126.16, 121.67, 62.54, 57.12.

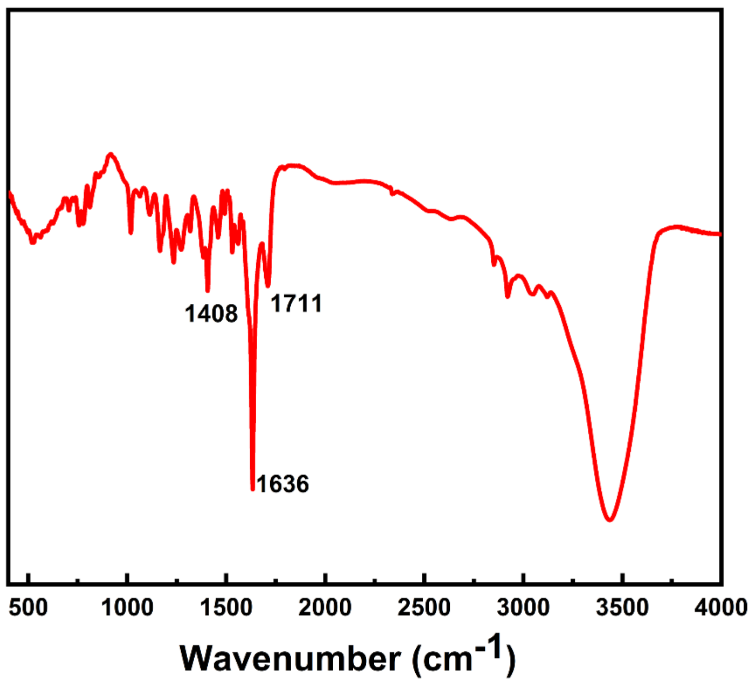

Figure S5. The FTIR spectrum of the Oxv ligand.

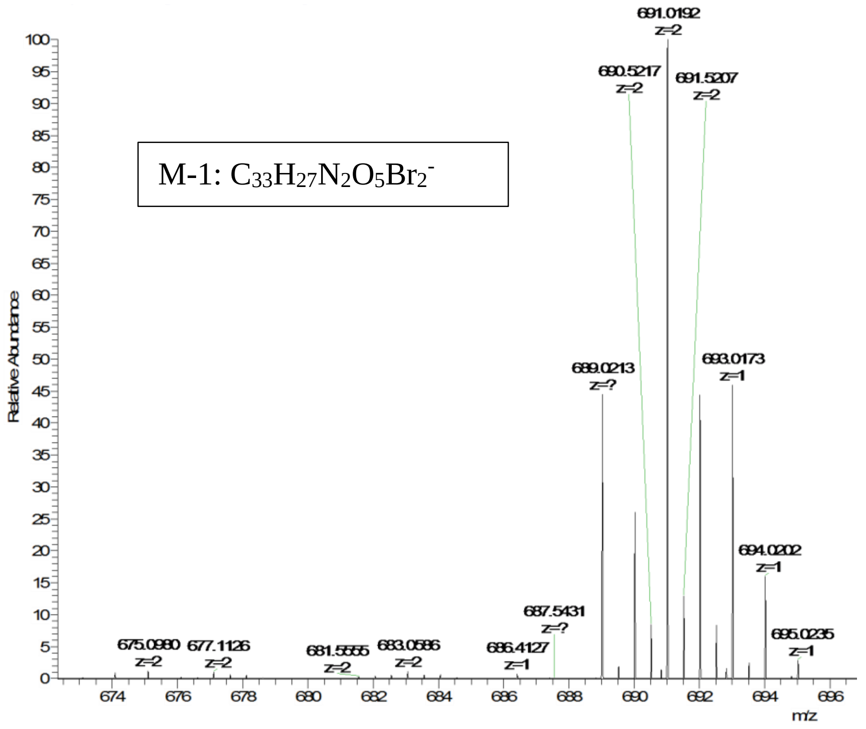

Figure S6. The MS spectrum of the Oxv ligand.

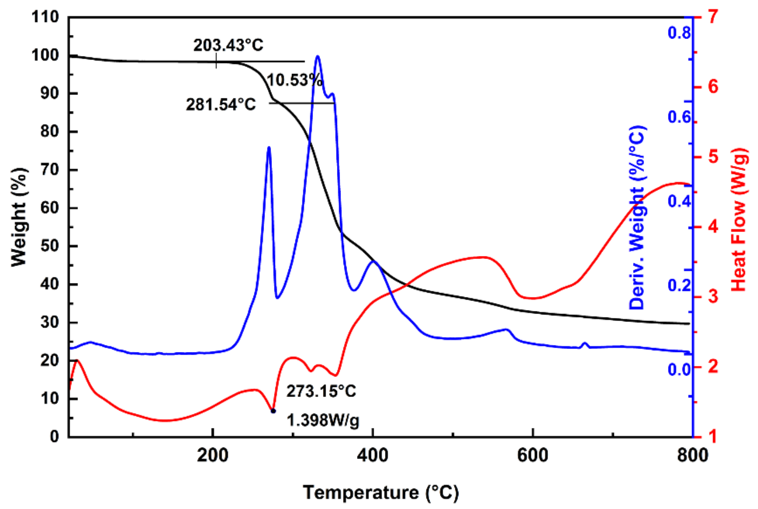

Figure S7. The TGA-DSC spectrum of the Oxv ligand.

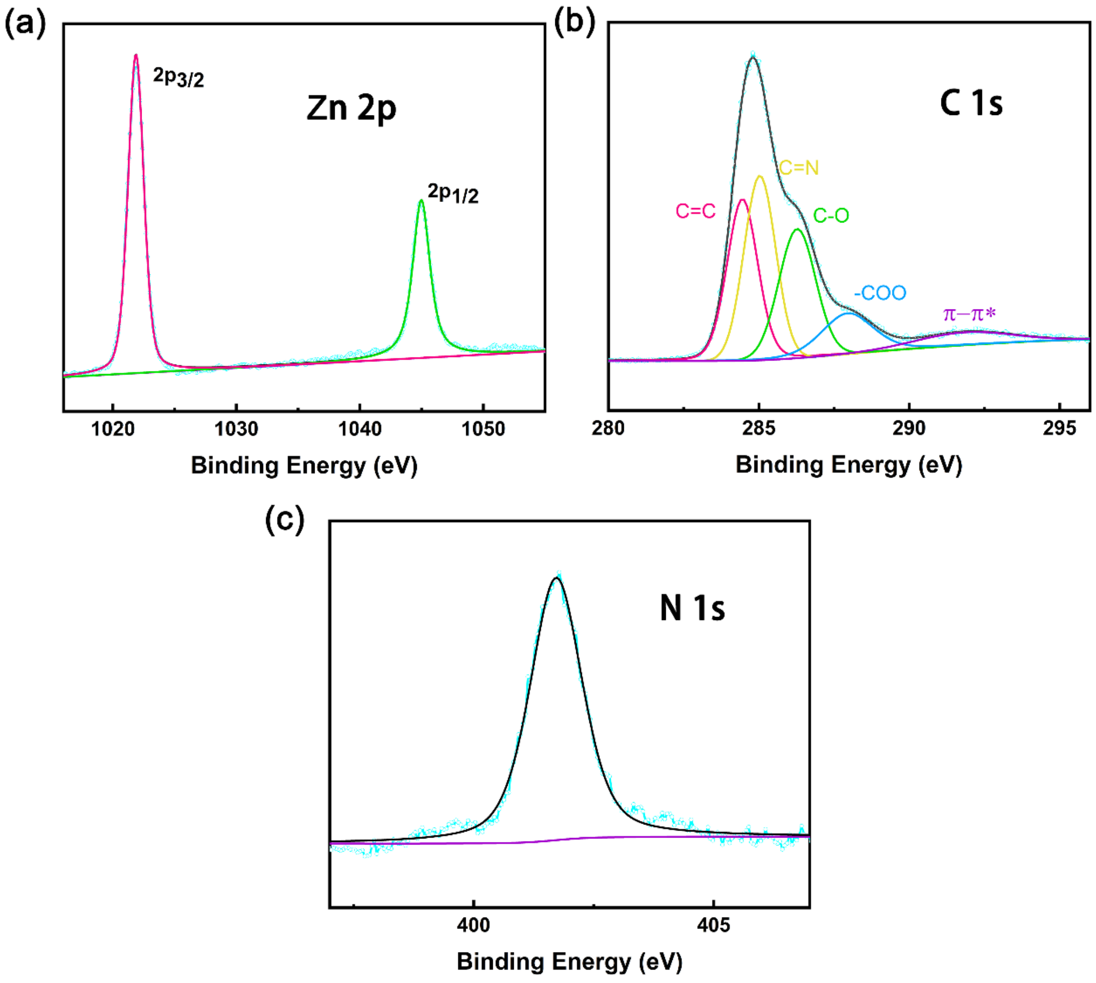

Figure S8. High-resolution XPS spectra of Zn 2p, C 1s and N 1s for the Zn-Oxv.

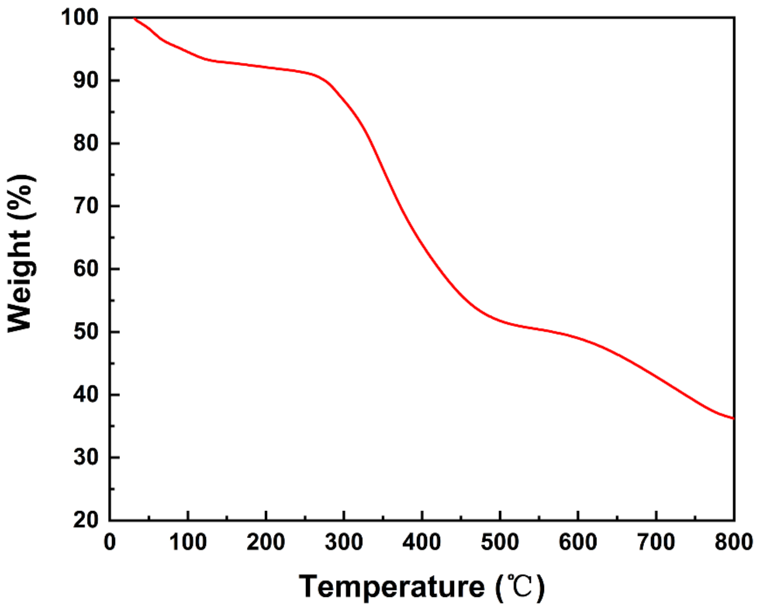

Figure S9. The TGA curve of Zn-Oxv CPs.

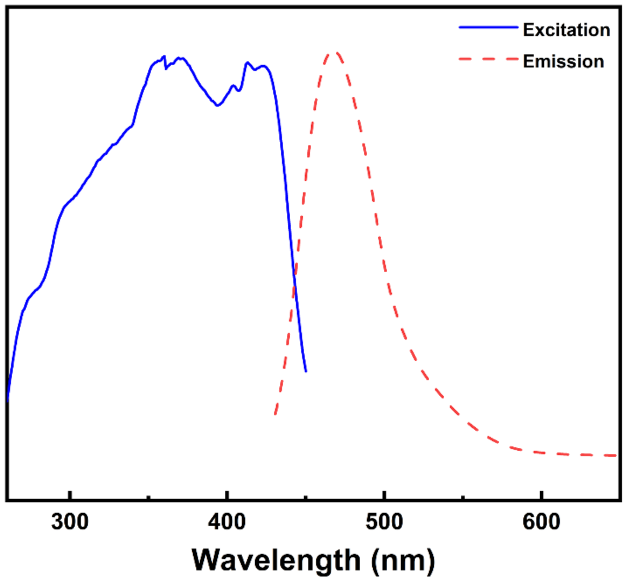

Figure S10. Fluorescence spectra of Zn-Oxv sample.

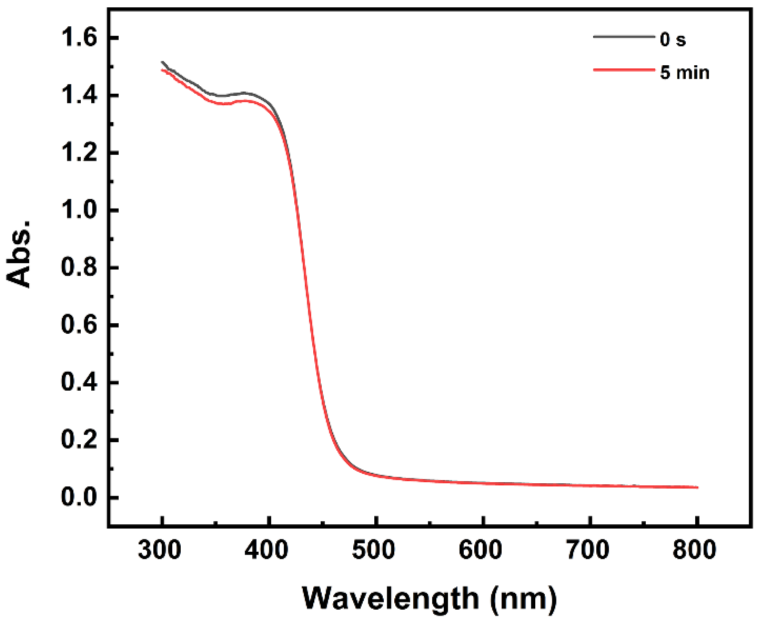

Figure S11. Solid-state UV−vis spectra of free ligand (Oxv) before and after irradiation.

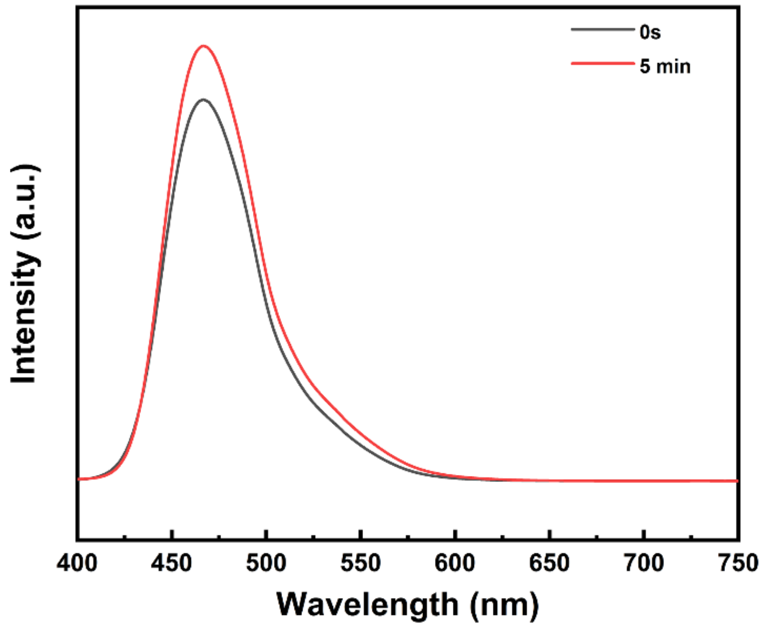

Figure S12. Emission spectra of free ligand (Oxv) before and after irradiation.

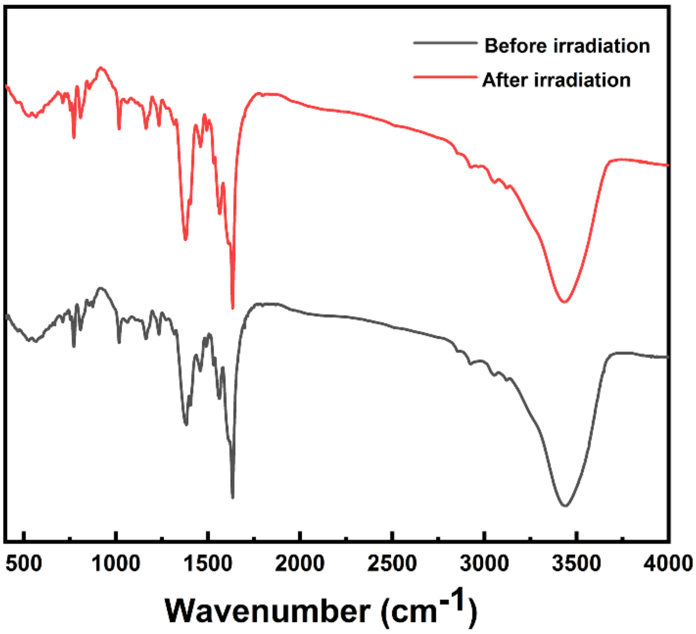

Figure S13. FTIR spectra of Zn-Oxv compound before and after irradiation.
